# Supplementary figures and images for: Integration of a neuronal RNAseq dataset with the draft Gryllus bimaculatus transcriptome refines gene predictions and highlights potential systematic response to injury
Source: bioRxiv. 2025 Jul 18:2025.07.13.663756. Preprint. [Version 1] doi: 10.1101/2025.07.13.663756 (PMC12338758; doi:10.1101/2025.07.13.663756)

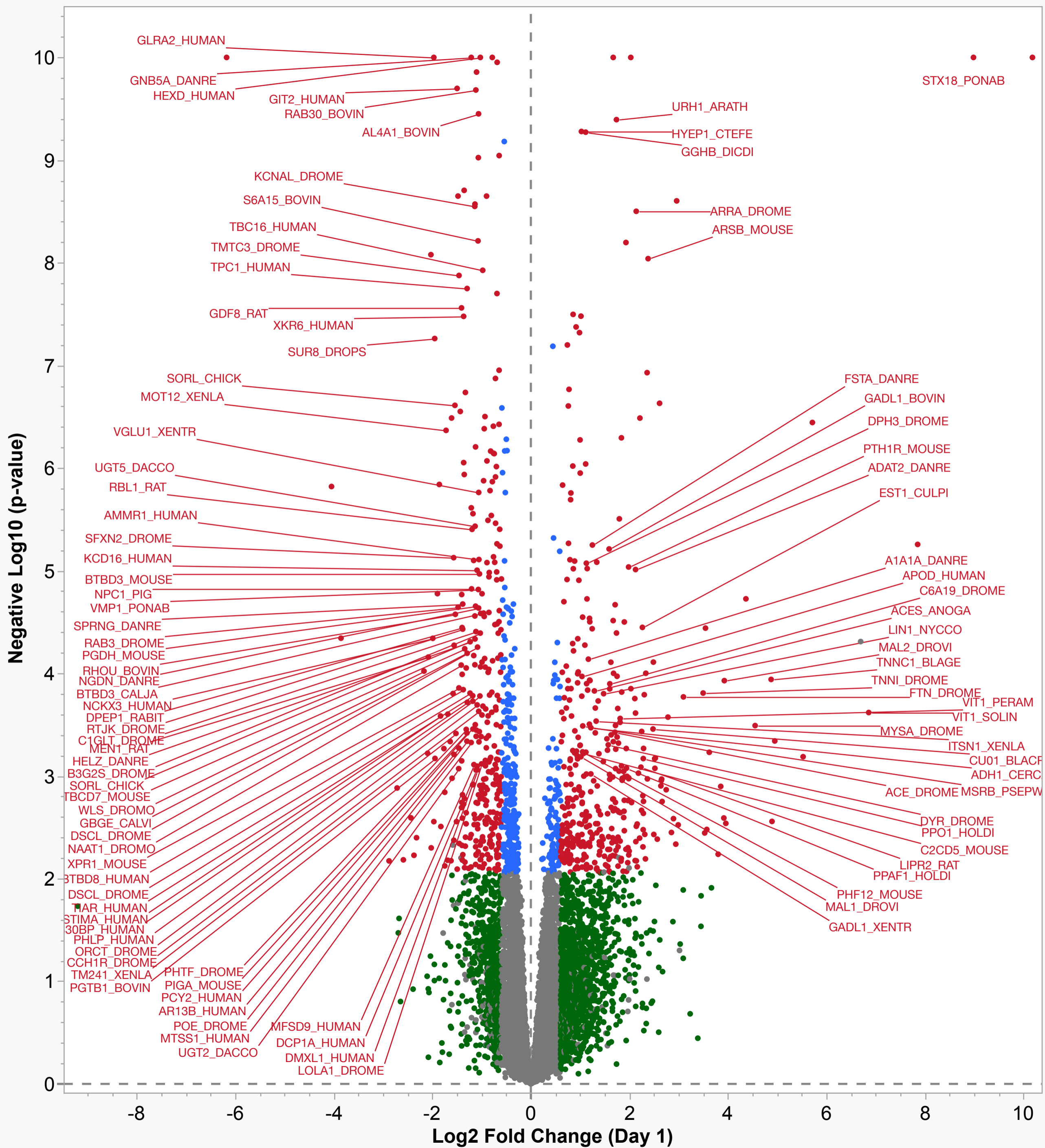

Supplement: Supplement 6 — S6 Figure: Volcano plots with selected genes labeled for A) Day 1, B) Day 3, and C) Day 7. [file media-6.pdf]

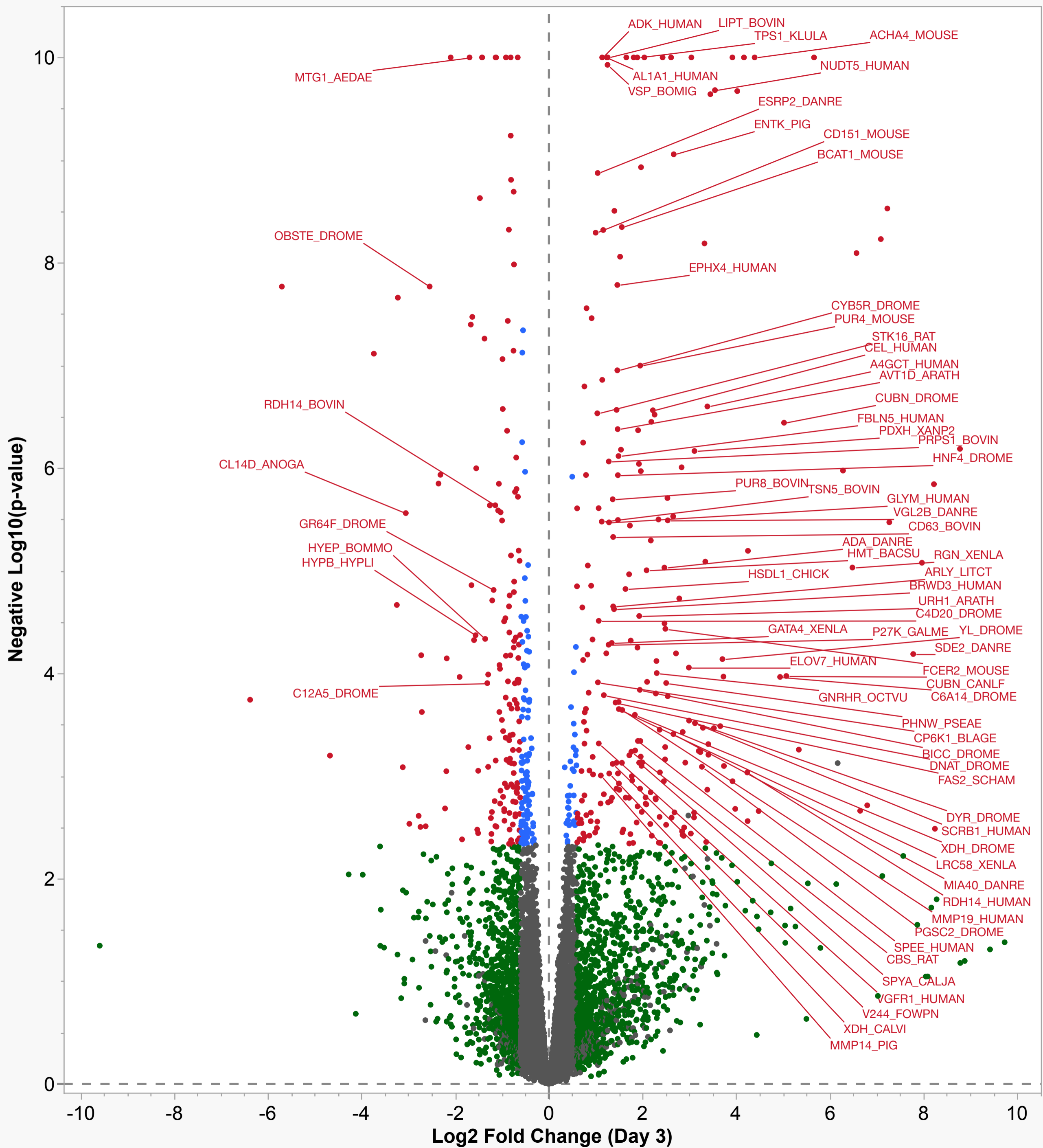

Supplement: Supplement 7 [file media-7.pdf]

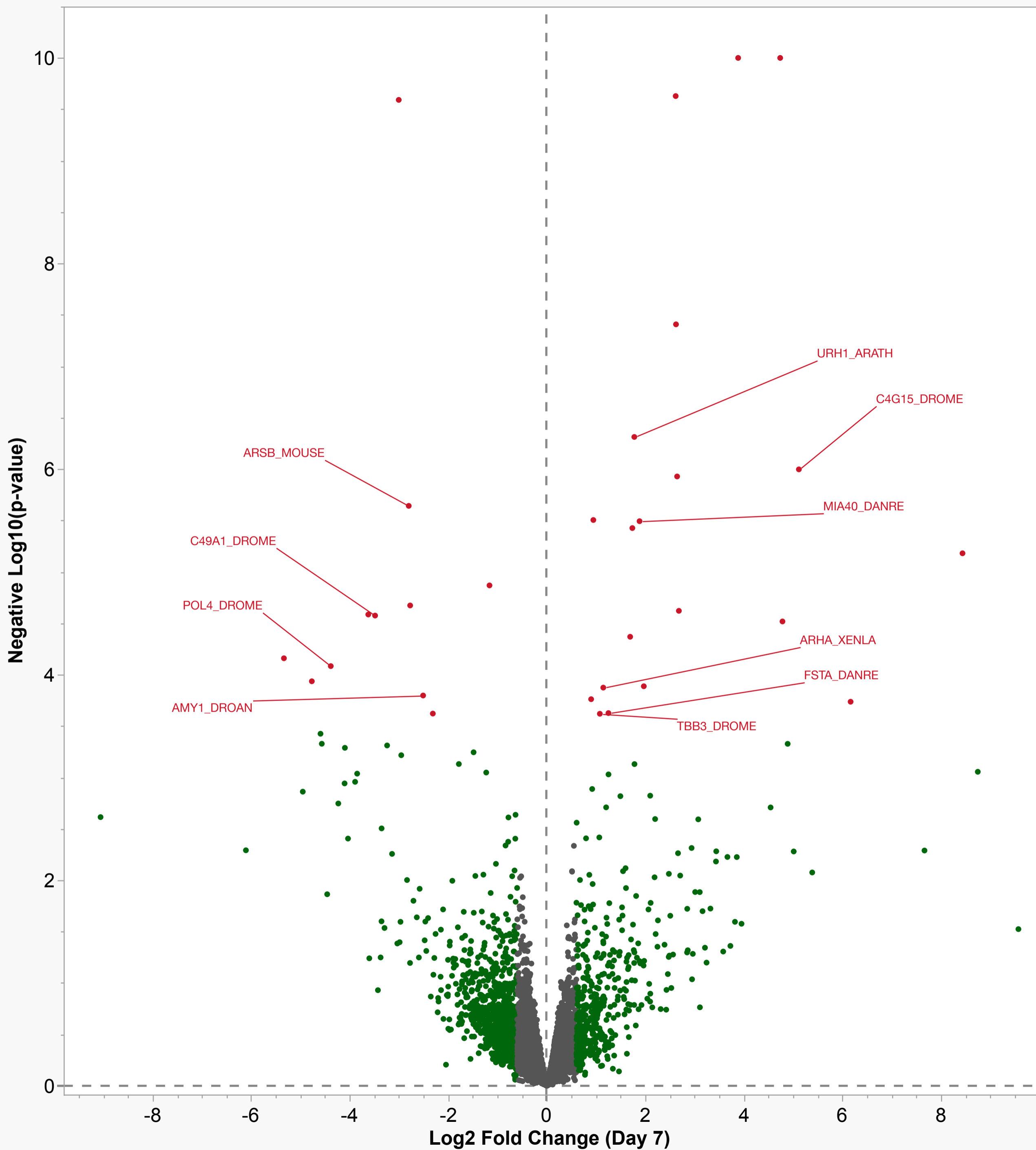

Supplement: Supplement 8 [file media-8.pdf]
